# Rapid increase in salivary IgA and broad recognition of spike protein following SARS-CoV-2 vaccination

**DOI:** 10.1016/j.virusres.2023.199294

**Published:** 2023-12-06

**Authors:** Kenji Ota, Hironori Sakai, Daisuke Sasaki, Fujiko Mitsumoto-Kaseida, Kei Sakamoto, Kosuke Kosai, Hiroo Hasegawa, Takahiro Takazono, Koichi Izumikawa, Hiroshi Mukae, Mya Myat Ngwe Tun, Kouichi Morita, Katsunori Yanagihara

**Affiliations:** a Department of Laboratory Medicine, Nagasaki University Hospital, 1-7-1, Sakamoto, Nagasaki 852-8501, Japan; b CellSpect Co. Ltd., 2-4, Kita Iioka, Morioka, Iwate 020-0857, Japan; c Department of Respiratory Medicine, Nagasaki University Hospital, 1-7-1, Sakamoto, Nagasaki 852-8501, Japan; d Infection Control and Education Center, Nagasaki University Hospital, 1-7-1, Sakamoto, Nagasaki 852-8501, Japan; e Department of Virology, Institute of Tropical Medicine (NEKKEN), Nagasaki University, 1-12-4, Sakamoto, Nagasaki 852-8102, Japan; f Dejima Infectious Disease Research Alliance, Nagasaki University, 1-12-4, Sakamoto, Nagasaki 852-8102, Japan

**Keywords:** SARS-CoV-2, Vaccine, Mucosal immunity, Saliva, IgA, IgG

## Abstract

Saliva is a key component of mucosal immunity, which protects the oral cavity from viral infections. However, salivary immune response to severe acute respiratory syndrome coronavirus 2 (SARS-CoV-2), in terms of immunoglobulin dynamics and recognition, have not been investigated sufficiently. In this study, saliva samples were collected from individuals that received SARS-CoV-2 vaccine, and immunoglobulin G (IgG), IgM, and IgA against whole spike protein and S1 protein were measured. IgA against whole spike protein increased significantly following vaccination, while IgA against S1 protein did not. Of note, the IgA response was evident two weeks after the first vaccine dose and continued to rise thereafter. On the contrary, IgG antibodies against S1 increased significantly at four weeks after vaccination. These results reveal the dynamics and recognition antigens of immunoglobulins in saliva, indicating the function of IgA in the mucosal immune system. These findings may pave the way for further studies on mucosal immune response induced by vaccination.

## Highlights

• We assessed salivary immune response to SARS-CoV-2 vaccine (BNT162b2; Pfizer).
• IgA against whole spike protein (WSP) increased 1 week after first vaccination.
• IgG against the S1 protein was elevated 4 weeks after first vaccination.
• IgG (WSP) titer was high at 8 weeks, booster response induced by 3rd vaccine dose.
• Rapid response of mucosal IgA provides first-line defense in infection prevention.

## Graphical abstract

**Figure:**
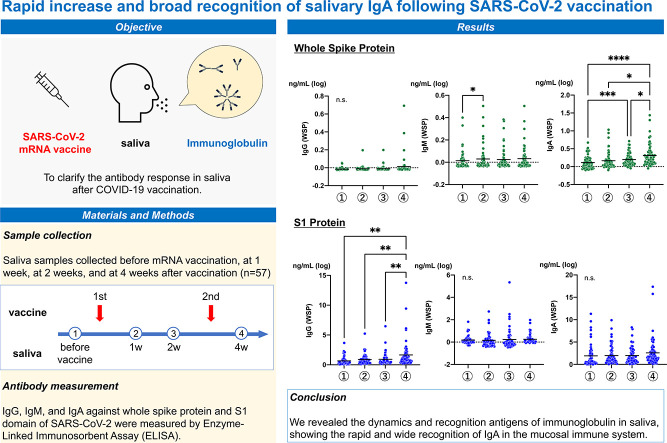

The antibody response induced by the severe acute respiratory syndrome coronavirus 2 (SARS-CoV-2) vaccine is well documented in blood samples, as an increase in immunoglobulin G (IgG) against the spike protein of SARS-CoV-2. It has been reported that IgG against spike protein correlates with neutralizing antibodies (Lustig et al., 2021), which indicates vaccine effectiveness in preventing infection and progression to severe disease (Khoury et al., 2021; Brosh-Nissimov et al., 2021). However, infection prevention relies on the immunity of the mucosal surface, through which the virus primarily intrudes into the host. Notably, the oral cavity is covered with mucosa, which acts as a portal for ingress and development of SARS-CoV-2 infection.

Saliva, produced in the oral cavity, is not only a cause of transmission via droplets, as it contains high viral loads (Ota et al., 2021; Li et al., 2020), but also plays a protective role against viruses, covering the oral cavity and assuming mucosal immunity (Russell et al., 2020). IgA plays a crucial role in mucosal immunity against SARS-CoV-2 (Havervall et al., 2022; Chan et al., 2021). However, studies assessing the dynamics and antigen recognition of immunoglobulin in the saliva of individuals who received SARS-CoV-2 mRNA vaccines are still limited.

A prospective cohort study was performed from March to April 2021 to assess the salivary immune responses to the SARS-CoV-2 vaccine among healthcare workers (HCWs) at Nagasaki University Hospital. The BNT162b2 vaccine (Pfizer, New York, NY, USA) was administered, which includes 30 µg of mRNA that encodes a Wuhan WT SARS-CoV-2 spike protein. All participants received two vaccine doses three weeks apart. In addition, the third dose of the vaccine was administered before 48 weeks after the first dose (between T7 and T8 as described below). Written informed consent was obtained from all the participants. This study was approved by the institutional review board of Nagasaki University Hospital (approval number: 21030301–4). IgG against the nucleocapsid protein (N) was measured using a chemiluminescence immunoassay (Alinity; Abbott Laboratories, Chicago, IL, USA) to exclude those with past natural SARS-CoV-2 infection. Saliva samples were obtained using Salivette™ (Sarstedt, Numbrecht, Germany) at four time points: before the first vaccine dose (T1) and one week (T2), two weeks (T3), and four weeks (T4) after the first vaccine dose (one week after the second dose) (Fig. 1A). Additionally, to conduct a longitudinal assessment of the antibody titer, saliva specimens were collected at 12 weeks (T5), 24 weeks (T6), 36 weeks (T7), and 48 weeks (T8) following vaccination. The samples were centrifuged at 3000 × g for 2 min and stored at −80 °C until antibody measurement. IgG, IgM, and IgA levels against the whole spike protein (WSP) and S1 domain (S1) of SARS-CoV-2 were measured by enzyme-linked immunosorbent assay (ELISA) using the following kits: coronavirus disease (COVID-19) Human IgM IgG ELISA kit (Spike protein); COVID-19 Human IgA ELISA kit (Spike protein, Full-Length) for WSP; COVID-19 Human IgM IgG ELISA kit (Spike protein, S1); and COVID-19 Human IgA ELISA kit (Spike protein, S1) (Cellspect Co., Ltd., Morioka, Japan) for S1. The mean antibody titer was measured as absorbance at an optical density (OD) of 450 nm. Consequently, we quantified the immunoglobulin titer using the standard as Anti-SARS-CoV-2 Spike S1 (8A5, human IgA; BioTimes, Irvine, CA, USA) for IgA, Fully Human RBD IgM Monoclonal Antibody (FPZ0589; Fapon Biotech, Guangdong, China) for IgM, and Fully Human RBD IgG Monoclonal Antibody (FPZ0570; Fapon Biotech, Guangdong, China) for IgG. The standard curves for each immunoglobulin were created by serial dilutions and approximation formulas were calculated. The antibody titer of each group was expressed as mean [95% CI] and compared across different time points using Tukey's multiple comparison test. Statistical significance was set at *P* < 0.05.

**Fig. 1:**
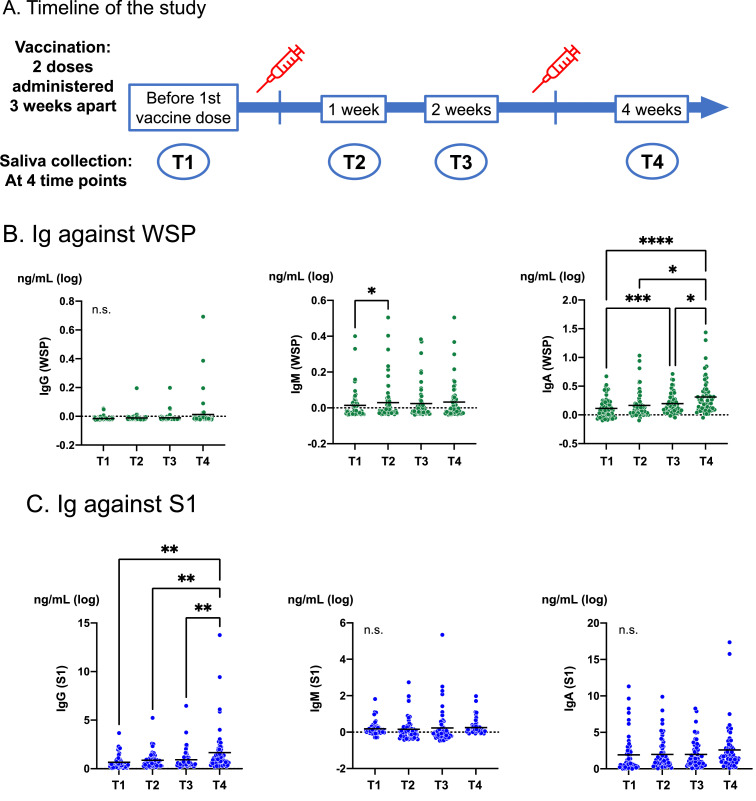
Timeline of the study and antibody titers of IgG, IgM, and IgA against the whole spike protein (WSP) and S1 protein. Fig 1A shows the timeline of the study. Saliva samples were collected before vaccine (T1), one week after 1st dose (T2), two weeks after 1st dose (T3), and four weeks after 1st dose (T4). Fig 1B shows each Ig titer against WSP, and Fig 1C shows Ig titer against S1. The antibody titers were measured by enzyme-linked immunosorbent assays at an optical density of 450 nm and expressed as ng/ml (log_10_). Bars represent means, and each dot represents individual sample data. Tukey's multiple comparison test was performed to assess the differences. Ig immunoglobulin. * *P* < 0.05, ** *P* < 0.01, *** *P* < 0.001, **** *P* < 0.0001. n.s. not significant.

A total of 57 HCWs were included in this study. None of the participants had positive results for IgG (N) or reported COVID-19 symptoms during the study period. The participants had a median age of 37.7 years and 28 (49.1%) were male. All patients tested negative for IgG (N) before the first vaccine dose; subsequently, saliva samples were collected. The antibody titers of each immunoglobulin against the target protein were assessed. For Ig against WSP (Fig 1B), no significant increase was observed in IgG or IgM through T1 to T4. Interestingly, IgA against WSP increased following vaccination (T1 1.423 ng/mL [95%CI: 1.235–1.611], T2 1.806 ng/mL [95%CI: 1.343–2.269], T3 1.725 ng/mL [95%CI: 1.499–1.951], and T4 2.938 ng/mL [95%CI: 1.840–4.035]), suggesting the reaction induced by vaccination. Notably, significant increase was observed two weeks after the first vaccine dose, followed by a continuous increase. For Ig against S1 (Fig 1C), IgA and IgM did not show an increase, but IgG increased significantly (T1 4.490 ng/mL [CI: 2.990–6.741], T2 7.454 ng/mL [CI: 4.509–12.331], T3 8.300 ng/mL [CI: 4.566–15.101], and T4 45.290 ng/mL [CI: 11.940–171.791]) after the second vaccination (T4). There were no correlations between IgA (WSP) and IgG (S1) levels for each individual. In addition, no difference in the antibody titers was observed between male and females. The antibody titers reached their peaks at T4, following which, the titer was maintained or gradually decreased for IgM (WSP), IgA (WSP), and all the Ig against S1 from T5 to T8 (Supplemental Fig. 1). However, for IgG (WSP), significant increase in titer was observed at T8 (T5 1.060 ng/mL [CI: 0.987–1.133], T6 1.031 ng/mL [CI: 0.993–1.069], T7 1.010 ng/mL [CI: 0.977–1.042], and T8 6.758 ng/mL [CI: 0.810–12.710]), suggesting booster response induced by the third dose of vaccination. Similar changes were not observed for the remaining antibodies, including IgG (S1).

In this study, we analyzed the antibody titers present in the saliva of participants who received SARS-CoV-2 mRNA vaccinations. A significant increase was observed in IgA against the WSP and in IgG against the S1 protein. IgA is the major immunoglobulin secreted in mucus and plays a crucial role in the immune function of the mucus membrane. A previous study measuring immunoglobulin in saliva reported that salivary IgA against the S1 protein did not show a significant increase in those without a history of infection (Azzi et al., 2022). Our results agree with this study's findings and further reveal that IgA against WSP was significantly increased in saliva. It can be inferred from our findings that vaccine-induced IgA exhibits broader recognition of the spike protein than IgG, indicating that IgA is ideal as a first-line defense. IgA against WSP increased one week after the first vaccination, whereas IgG against the S1 protein was elevated four weeks after the first vaccination. Consistent with this finding, Chan et al. reported an earlier increase in S1-specific IgA compared with that of IgG in the nasal epithelial-lining fluid after administrating the mRNA vaccine (Chan et al., 2021). This rapid response of mucosal IgA supports its important role as a first-line defense in preventing infection of the mucosal membrane. A limitation of this study is that we could not confirm the neutralization activity of saliva antibodies against SARS-CoV-2. We performed the neutralization assay using several saliva samples with high antibody titers, including five samples for IgA (WSP) and four samples for IgG (S1). However, none of the samples showed any neutralization. Therefore, we assumed that the saliva antibody titers were not correlated with neutralization, and we did not test the rest of the samples in this study for this outcome. In the previous study that assessed the immunoglobulin and neutralization in serum and saliva, only 18% of saliva obtained from those without previous infection showed neutralization activity (Azzi et al., 2022). In that study, both IgA and IgG in saliva showed lower titer than those in serum. Therefore, it is speculated that immunoglobulin secreted in saliva is not sufficient to effect neutralization. Another limitation is that the impact of previous SARS-CoV-2 infection has not yet been investigated. Nevertheless, the results in this study clarify the recognition antigens and antibody dynamics in saliva, which provides novel insights to elucidate mucosal immunity induced by vaccination.

In conclusion, we observed a rapid increase in IgA against WSP in the saliva obtained from individuals vaccinated for SARS-CoV-2. This is the first study revealing the dynamics and recognition antigens of IgA in saliva and highlighting the function of IgA in the mucosal immune system. Our findings can inspire further research into the clarification on mucosal immunity induced by vaccination.

**Supplementary Fig.** Antibody titers of IgG, IgM, and IgA against the whole spike protein (A) and S1 protein (B) obtained at 12 weeks (T5), 24 weeks (T6), 36 weeks (T7), and 48 weeks (T8) following vaccination. The antibody titers were measured by enzyme-linked immunosorbent assays at an optical density of 450 nm and expressed as ng/ml (log_10_). Bars represent means, and each dot represents individual sample data. Tukey's multiple comparison test was performed to assess the differences. Ig immunoglobulin. * *P* < 0.05, ** *P* < 0.01, *** *P* < 0.001, **** *P* < 0.0001. n.s. not significant

## CRediT authorship contribution statement

**Kenji Ota:** Conceptualization, Data curation, Formal analysis, Funding acquisition, Investigation, Software, Validation, Visualization, Writing – original draft, Writing – review & editing. **Hironori Sakai:** Data curation, Methodology, Writing – review & editing. **Daisuke Sasaki:** Methodology, Writing – review & editing. **Fujiko Mitsumoto-Kaseida:** Supervision, Writing – review & editing. **Kei Sakamoto:** Supervision, Writing – review & editing. **Kosuke Kosai:** Supervision, Writing – review & editing. **Hiroo Hasegawa:** Supervision, Writing – review & editing. **Takahiro Takazono:** Supervision, Writing – review & editing. **Koichi Izumikawa:** Supervision, Writing – review & editing. **Hiroshi Mukae:** Supervision, Writing – review & editing. **Mya Myat Ngwe Tun:** Methodology, Writing – review & editing. **Kouichi Morita:** Methodology, Supervision. **Katsunori Yanagihara:** Conceptualization, Project administration, Resources, Software, Supervision, Writing – review & editing.

## Declaration of Competing Interest

The authors declare that they have no known competing financial interests or personal relationships that could have appeared to influence the work reported in this paper.

## Data Availability

Data will be made available on request.

## Appendix Supplementary materials

Image, application 1
