# Supplementary material for: Rapid increase in salivary IgA and broad recognition of spike protein following SARS-CoV-2 vaccination
Source: Virus Res. 2023 Dec 6;339:199294. doi: 10.1016/j.virusres.2023.199294 (PMC10749273; doi:10.1016/j.virusres.2023.199294)
Supplement: Supplementary file 1 [file mmc1.pptx]

## Slide 1
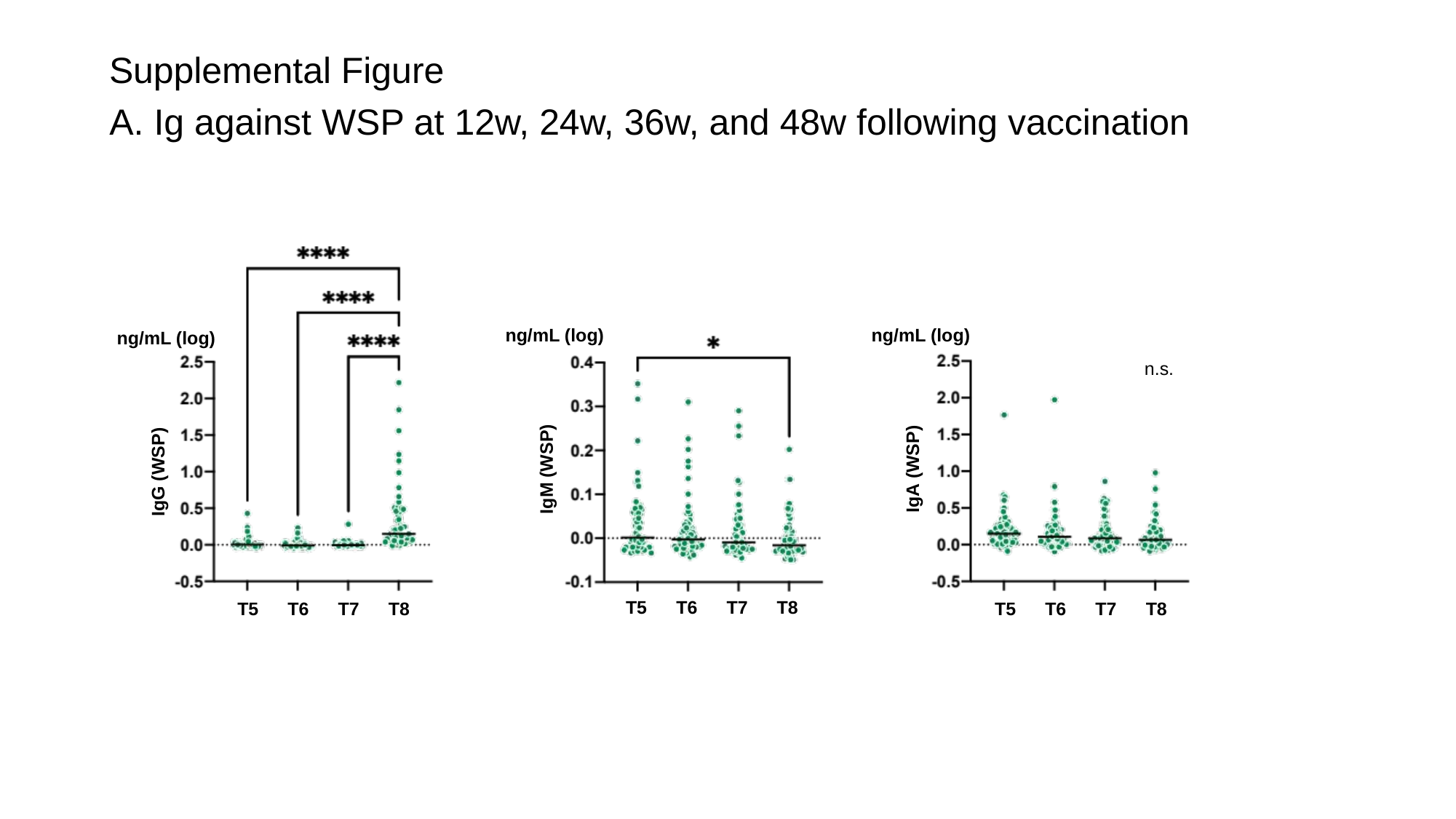

Supplemental Figure
A. Ig against WSP at 12w, 24w, 36w, and 48w following vaccination
ng/mL (log)
ng/mL (log)
ng/mL (log)
n.s.
IgM (WSP)
IgA (WSP)
IgG (WSP)
| T5 | T6 | T7 | T8 |
| --- | --- | --- | --- |
| T5 | T6 | T7 | T8 |
| --- | --- | --- | --- |
| T5 | T6 | T7 | T8 |
| --- | --- | --- | --- |

## Slide 2
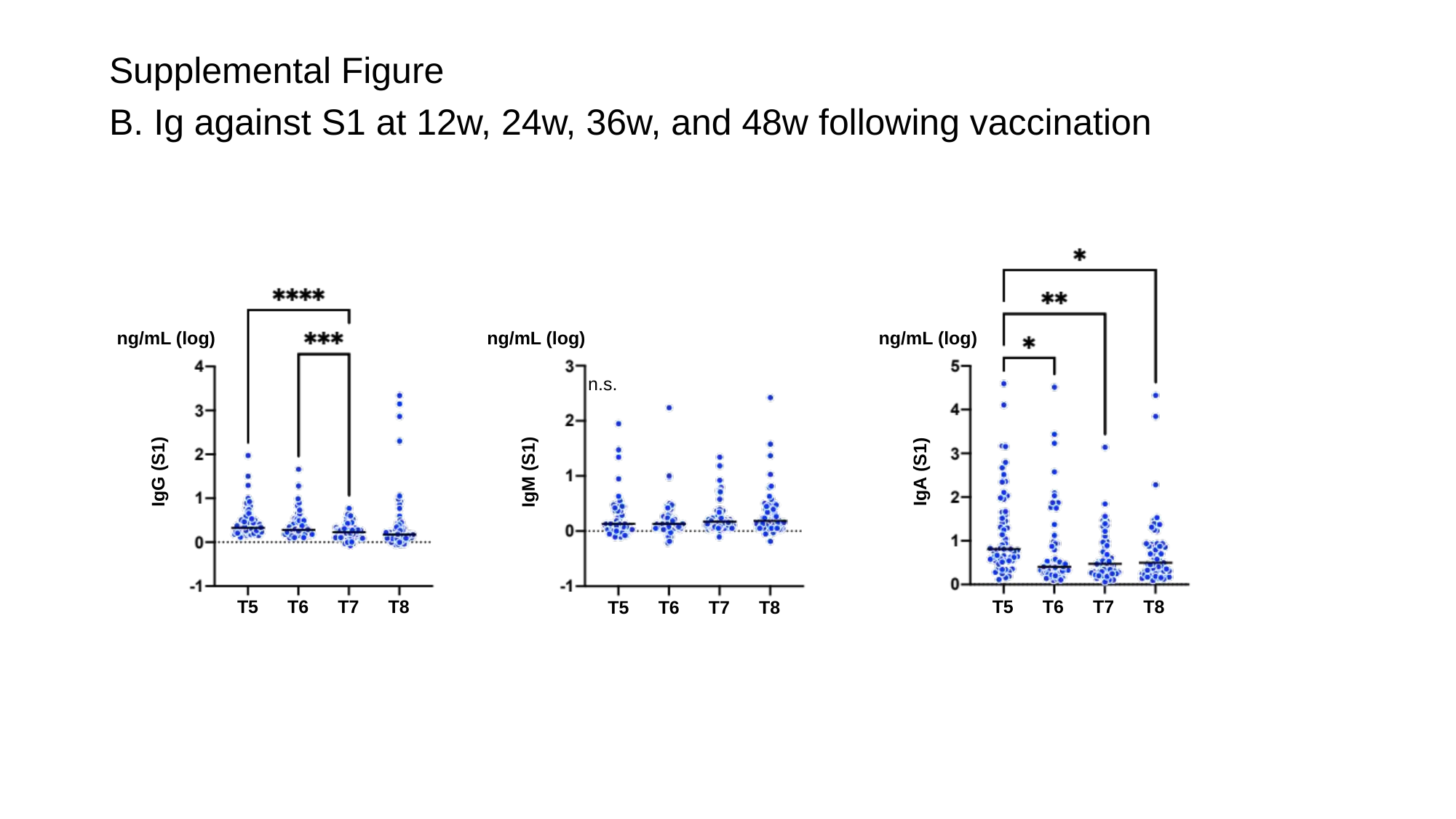

Supplemental Figure
B. Ig against S1 at 12w, 24w, 36w, and 48w following vaccination
ng/mL (log)
ng/mL (log)
ng/mL (log)
n.s.
IgG (S1)
IgM (S1)
IgA (S1)
| T5 | T6 | T7 | T8 |
| --- | --- | --- | --- |
| T5 | T6 | T7 | T8 |
| --- | --- | --- | --- |
| T5 | T6 | T7 | T8 |
| --- | --- | --- | --- |
